# Mucosal-Associated Invariant T Cell Interactions with Commensal and Pathogenic Bacteria: Potential Role in Antimicrobial Immunity in the Child

**DOI:** 10.3389/fimmu.2017.01837

**Published:** 2017-12-15

**Authors:** Liana Ghazarian, Sophie Caillat-Zucman, Véronique Houdouin

**Affiliations:** ^1^INSERM UMR1149, Centre de Recherche sur l’Inflammation, Université Paris Diderot, Paris, France; ^2^Laboratoire d’Immunologie, Hôpital Saint Louis, AP-HP, Paris, France; ^3^Service des Maladies Digestives et Respiratoires de l’Enfant, Hôpital Robert Debré, AP-HP, Paris, France

**Keywords:** mucosal-associated invariant T cells, invariant T cells, innate immunity, antimicrobial defense, riboflavin

## Abstract

Mucosal-associated invariant T (MAIT) cells are unconventional CD3^+^CD161^high^ T lymphocytes that recognize vitamin B2 (riboflavin) biosynthesis precursor derivatives presented by the MHC-I related protein, MR1. In humans, their T cell receptor is composed of a Vα7.2-Jα33/20/12 chain, combined with a restricted set of Vβ chains. MAIT cells are very abundant in the liver (up to 40% of resident T cells) and in mucosal tissues, such as the lung and gut. In adult peripheral blood, they represent up to 10% of circulating T cells, whereas they are very few in cord blood. This large number of MAIT cells in the adult likely results from their gradual expansion with age following repeated encounters with riboflavin-producing microbes. Upon recognition of MR1 ligands, MAIT cells have the capacity to rapidly eliminate bacterially infected cells through the production of inflammatory cytokines (IFNγ, TNFα, and IL-17) and cytotoxic effector molecules (perforin and granzyme B). Thus, MAIT cells may play a crucial role in antimicrobial defense, in particular at mucosal sites. In addition, MAIT cells have been implicated in diseases of non-microbial etiology, including autoimmunity and other inflammatory diseases. Although their participation in various clinical settings has received increased attention in adults, data in children are scarce. Due to their innate-like characteristics, MAIT cells might be particularly important to control microbial infections in the young age, when long-term protective adaptive immunity is not fully developed. Herein, we review the data showing how MAIT cells may control microbial infections and how they discriminate pathogens from commensals, with a focus on models relevant for childhood infections.

## Introduction

T lymphocytes are mainly categorized into either conventional CD4 or CD8 T cells, or unconventional invariant T cells. In conventional T cells, combinations of T cell receptor (TCR) α and β chains are unlimited and adapted for optimal T cell responses to numerous types of pathogens, whereas those of unconventional T cells are much more limited and suited for innate-like immunity. Together with this limited diversity, unconventional T cells are restricted by non-classical MHC molecules, while conventional T cells recognize classical MHC/peptide complexes. In humans, mucosal-associated invariant T (MAIT) cells represent the most abundant semi-invariant αβT cell subset (1–3). MAIT cells are preferentially localized in mucosal tissues and react against a newly identified class of microbial-derived antigen precursors presented by the non-classical MHC-I-related molecule, MR1. Upon microbial infection, MAIT cells rapidly produce cytokines and cytotoxic effectors. MAIT cells are protective in experimental models of infection and are decreased in the blood of patients with bacterial infections. Here, we review the rapidly evolving field of the protective role of MAIT cell in infectious diseases, with a particular emphasis on models that may be of special interest in children.

## Main Characteristics of MAIT Cells

Mucosal-associated invariant T cells represent an abundant proportion of resident T cells in tissues (20–40% in the liver, 1–8% in the colon lamina propria, and 10–20% in the lung and female genital tract) (4–10). They also represent 1–10% of the entire CD3 T cell pool in human peripheral blood (7, 11). This compares with around 0.1% for invariant Natural Killer T (iNKT) cells, another population of unconventional innate-like T cells. In contrast, MAIT cells are 10-fold less abundant in mice than in humans and iNKT are more numerous. MAIT cells express a semi-invariant TCR made of a canonical TCRα chain (Vα7.2-Jα33/20/12 in humans, Vα19-Jα33 in mice) paired with a limited number of TCRβ chains (12–15). The MAIT TCR recognizes the conserved, monomorphic, MHC class I-related molecule, MR1 (10), which binds riboflavin (vitamin B2) biosynthesis precursor derivatives, such as 5-(2-oxopropylideneamino)-6-d-ribitylaminouracil (5-OP-RU) produced by most, but not all, bacteria and yeasts (16, 17). MAIT cell activation requires key genes encoding enzymes that form an early intermediate (5-A-RU) in bacterial riboflavin synthesis. Although 5-A-RU does not bind MR1 or activate MAIT cells directly, it forms potent MAIT-activating antigens *via* non-enzymatic reactions with distinct host- or bacteria-derived small chemical molecules, such as glyoxal and methylglyoxal, derived from other metabolic pathways (16, 17). This represents a unique mechanism for creating T-cell ligands from disparate metabolite building blocks. A wide range of bacteria and fungi, but not mammalian cells or viruses, are able to synthesize riboflavin and hence provide MR1 ligands (7, 11, 17). Thus, only microbes that possess a riboflavin biosynthetic pathway have a direct, MR1-dependent, MAIT-activating capacity. Certain bacteria, including *Enterococcus faecalis, Listeria monocytogenes*, and group A *Streptococcus* do not activate MAIT cells, likely due to the lack of an intact riboflavin biosynthetic pathway in these strains (7). As humans do not synthesize riboflavin, the MR1–MAIT axis accordingly represents a sophisticated discriminatory mechanism for targeting microbial antigens while protecting the host.

The vast majority of human MAIT cells are CD8^+^, although some CD4^+^ and double-negative CD4^−^CD8^−^ MAIT subsets are also detected (2, 14, 18). In addition, MAIT cells express high levels of the C-type lectin CD161 and IL-18 receptor α (IL-18Rα) (7, 11, 19). Recently, they have become easily identifiable in the peripheral blood by MR1 tetramers loaded with the bacterial ligand 5-OP-RU (available from the NIH tetramer facility) (14). MAIT cells also express the CXCR6 and CCR9 chemokine receptors, which are involved in trafficking to peripheral tissues, especially the intestine and liver (4, 10, 20) but do not express CCR7, involved in migration to lymph nodes. Like iNKT cells, MAIT cells express the master promyelocytic leukemia zinc finger transcription factor (PLZF), suggesting a common thymic differentiation program (3, 21). They also express RORγ, Tbet, Helios, and Eomes (22), consistent with their various effector functions.

**Figure 1:**
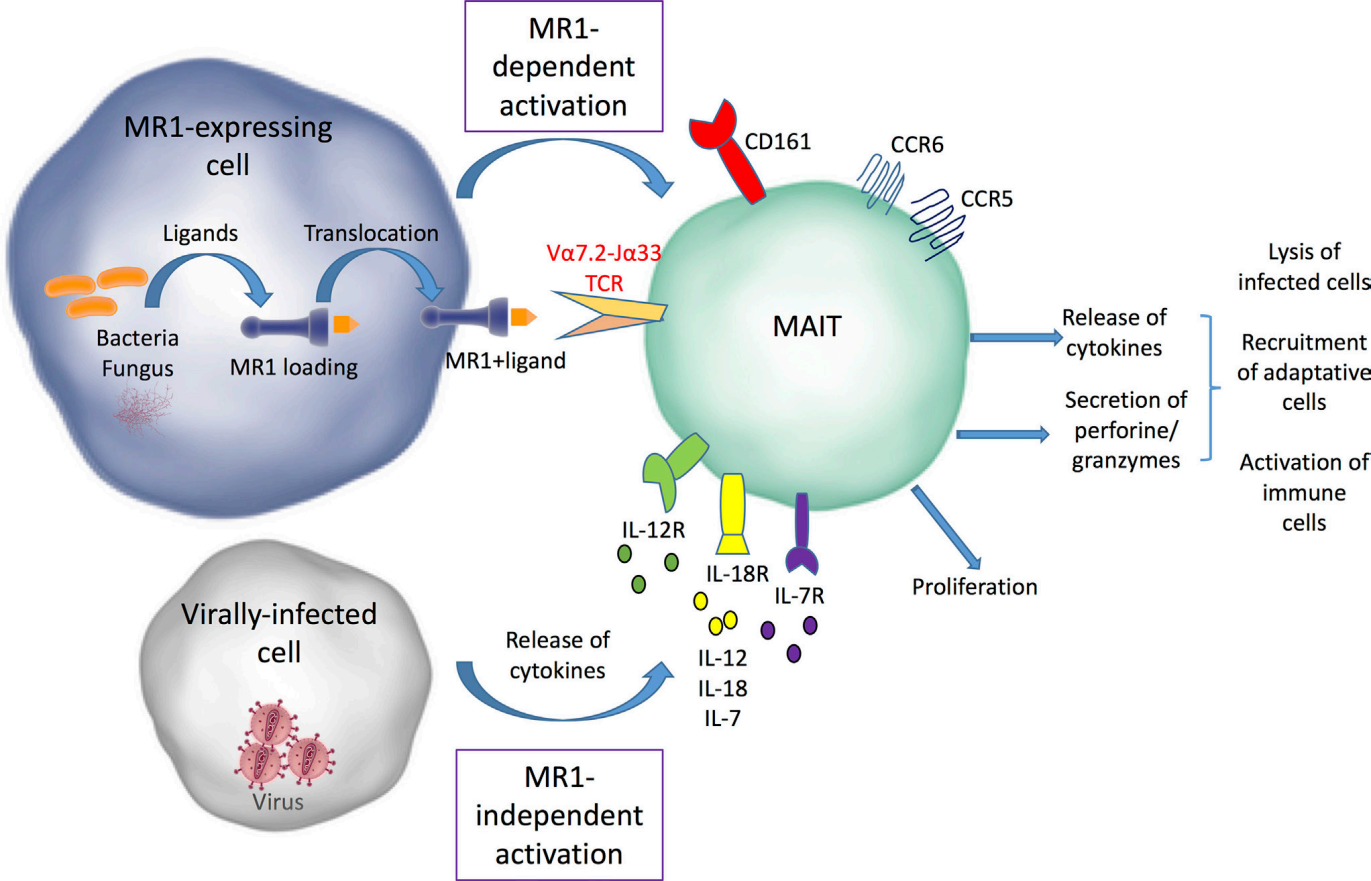
MR1-dependent and independent mucosal-associated invariant T (MAIT) cell activation. Bacterial and fungal ligands can be presented by MR1 to MAIT cells and induce their activation. MAIT cells can also be activated independently from MR1 by different types of cytokines secreted by infected cells. After their activation, MAIT cells proliferate and release cytokines and cytolytic enzymes, which allow infected cell lysis and promote the recruitment and activation of other immune cells.

Upon TCR-dependent recognition of microbial antigens, MAIT cells display immediate effector responses, by secreting inflammatory cytokines (IFNγ, TNF-α, IL-17, and sometimes IL-22) and mediating perforin-dependent cytotoxicity against bacterially infected cells (7, 11, 20, 23, 24) (Figure 1). This strongly supports their involvement in antimicrobial defense. Cytokines produced by MAIT cells may not only act directly on infected target cells, but also promote activation of other immune cells and orchestrate adaptive immunity through dendritic cell (DC) maturation (25, 26). Importantly, human MAIT cells can also be activated *in vitro* in a TCR-MR1 independent fashion in response to cytokines such as IL-12, IL-18, IL-15, and/or interferon α/β (27–29). Consequently, MAIT cells can be activated in various non-bacterial inflammatory conditions in which these cytokines are produced, in particular during acute or chronic viral infections such as dengue, influenza virus, HCV, and HIV (28, 30–34). For the same reasons, MAIT cells may participate in non-infectious pathological conditions, such as autoimmune disorders and cancer [for review, see Ref. (35–37)].

Finally, in addition to microbial products derived from vitamin B2 synthesis, other MR1-binding ligands have been identified, including the non-stimulatory folic acid (vitamin B9) derivative 6-formyl-pterin (6-FP) (17), and various activating and non-activating drugs and drug-like molecules (38). So far, the clinical relevance of these ligands is yet to be elucidated.

## MAIT Cell Development

MAIT cells are selected on MR1-expressing CD4^+^CD8^+^ thymocytes (39) and exit the thymus with a naïve phenotype before acquiring memory characteristics and expanding in the periphery (4, 18). As recently demonstrated using MR1 tetramers, the intrathymic development of MAIT cells is divided into three stages defined by expression of CD161 and CD27. Immature stage 1 and stage 2 MAIT cells (CD161^−^ in the human) predominate in thymus but represent minor subsets in periphery, where mature stage 3 MAIT cells (CD161^high^) are largely predominant. In germ-free mice, immature stage 1 MAIT cells are generated in the thymus but mature MAIT cells are absent from the periphery, which suggests that colonization by the commensal microbiota provides a key maturation signal. Indeed, colonization of the gut with even a single type of bacteria, capable of providing a ligand for MR1, is enough to restore the normal development of both thymic and peripheral MAIT cells (10, 18, 40).

**Figure 2:**
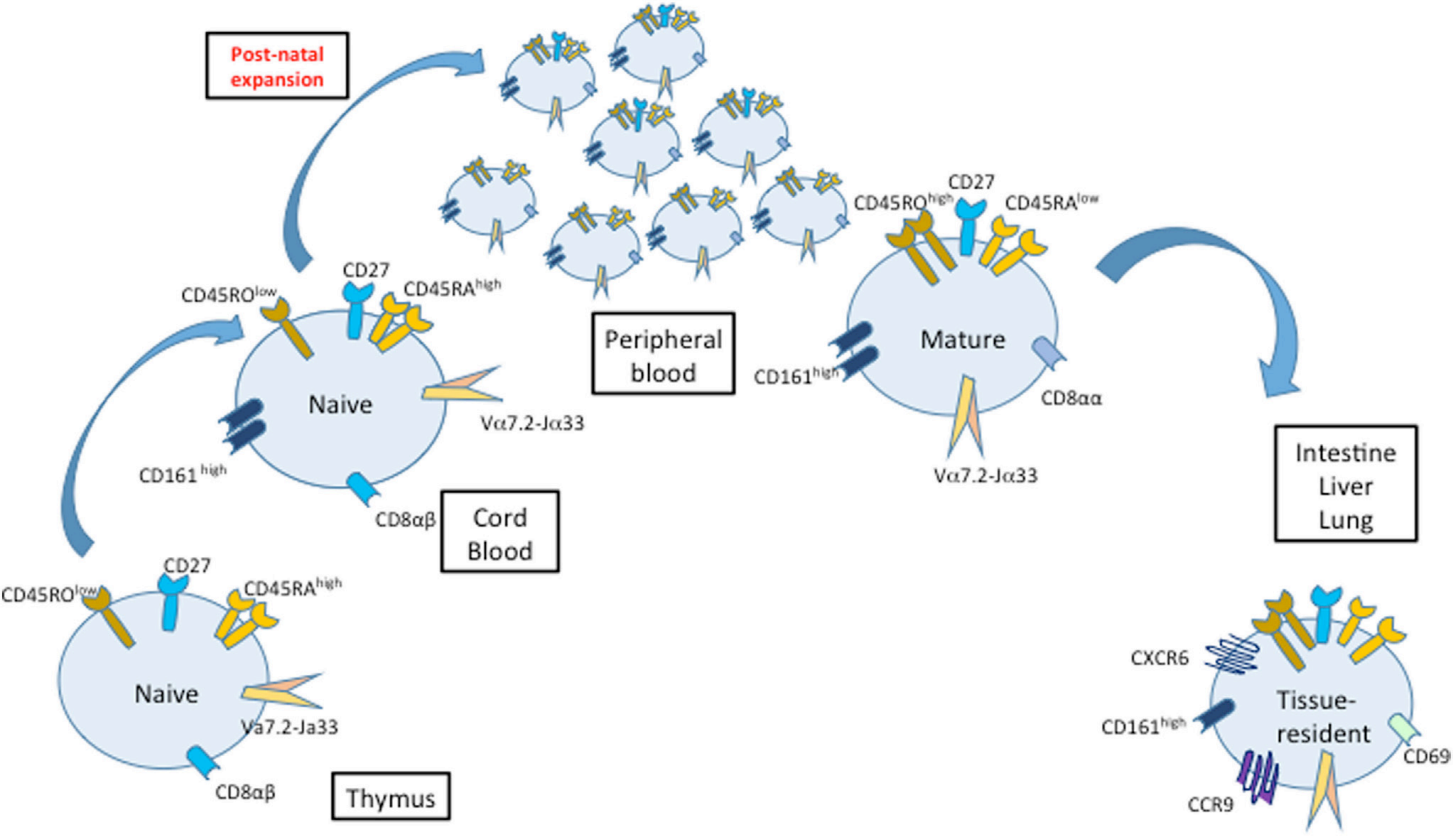
Maturation, proliferation, and migration of mucosal-associated invariant T (MAIT) cells. MAIT cells have a naive phenotype (CD45RA^high^/CD27/CD45RO^lo^) and a low frequency in cord blood compared with adult peripheral blood. Classically, MAIT cell development in the thymus is divided into three stages defined by their expression of CD161 and CD27, with stage 3 MAIT cells resembling MAIT cells found in peripheral blood after birth. After the birth, MAIT cells proliferate and acquire a mature phenotype. The colonization of different organs starts already during fetal development.

At birth, cord blood MAIT cells are naïve and represent a very small proportion of T cells (less than 0.1% of T cells), while they are predominant and exhibit mature characteristics in adult peripheral blood (4, 18, 20, 40) (Figure 2). This indicates that MAIT cell thymopoiesis is complemented by an important postnatal peripheral expansion. Surprisingly, mature tissue-resident MAIT cells are detected in the intestine, lung, and liver (but not in the spleen and mesenteric lymph nodes) of second trimester human fetuses (41). The nature of MR1 ligands present during fetal life remains elusive, as it is believed that the fetus *in utero* is sterile and that colonization with microorganisms starts only after birth. Nevertheless, even in the absence of live microbes in the placenta, maternal gestational commensals may be a source of diffusible metabolites reaching fetal tissues (42). Moreover, recent studies indicate that microbial colonization already occurs *in utero* (43, 44). Therefore, early interactions with the maternal or fetal microbiome may influence MAIT cell development, as suggested for other immune cell subsets (45).

The mechanisms driving postnatal MAIT cell expansion in the human remain unclear. In mice, the presence of B cells is necessary for peripheral expansion of MAIT cells but not for their thymic selection (18). In patients with common variable immunodeficiency, some of whom have undetectable circulating B cells, MAIT cell frequencies are decreased, but there is no association between MAIT cell and B cell frequencies (46). It is likely that even a small number of B cells in the lamina propria are sufficient for driving peripheral MAIT cell expansion, as shown in mice with a transmembrane immunoglobulin-μ mutation, in which B lymphocytes are absent in the peripheral blood, but some immunoglobulin A-producing B lymphocytes are found in the intestine (10). This may suggest that the initial proliferation of MAIT cells occurs close to the intestine where bacterial-derived MR1 ligands are abundant. MAIT cells do not expand in mice lacking MR1 in the periphery, or in mice colonized with bacteria lacking MR1 ligand (7). Furthermore, variable microbe-mediated expansion of peripheral MAIT cells was demonstrated in different mouse models, in particular a tremendous expansion during *Francisella tularensis* infection (24). Taken together, these observations indicate that peripheral MAIT cell expansion is likely dependent on encounters with microbial-derived ligands, although this remains difficult to demonstrate in humans. Few studies showed that MAIT cell frequencies gradually increase with age in the peripheral blood of healthy children (4, 40). Moreover, MAIT cell numbers exhibit very large interindividual variability (over one log range) in the blood of both children and adults, but the relationship with previous infections has never been documented. It is tempting to speculate that the peripheral development of MAIT cells follows a two-step program, in which early interactions with the commensal microbiota provide a first maturation signal, followed by variable MAIT cell expansion related to encounters with different microbes. Only a careful longitudinal analysis of MAIT cells levels in children of various ages with documented microbial infection history will confirm this hypothesis.

## Antimicrobial Function of MAIT Cells

*In vitro*, MAIT cells are activated in the presence of MR1-expressing cells loaded with bacterial preparations (fixed *Escherichia coli* and *Mycobacterium tuberculosis* lysate) or cells experimentally infected with various strains of bacteria and yeasts (*E. coli, Salmonella typhimurium, M. tuberculosis*, and *Candida albicans*). Such activated MAIT cells produce inflammatory cytokines and cytolytic molecules (7, 11, 23, 27, 47) and can kill infected epithelial cells (22, 23, 27). MAIT cells are also able to inhibit *Mycobacterium bovis* bacillus Calmette–Guérin (BCG) growth in infected macrophages (12, 48), suggesting that they may control microbial burden *in vivo*.

A better understanding of the role of MAIT cells in the control of microbial infections has been obtained through several mouse models, in particular transgenic mice overexpressing MAIT cells and MR1-deficient mice, compared with wild-type mice. In MAIT transgenic mice, activated MAIT cells accumulate at the site of *E. coli* or *Mycobacterium abscessus* infection and promote bacterial clearance, except if mice are MR1-deficient (7). A low dose of *M. bovis* aerosol results in a much stronger infection in MR1-deficient mice compared with control mice, indicating the important role of MAIT cells in the early control of mycobacterial infection in the lung (48). An increase in bacterial load is also observed in MAIT-deficient mice infected with *Klebsiella pneumonia* (49). MAIT cells accumulate in the lung of mice after intranasal inoculation of *S. typhimurium* (50). The contribution of MAIT cells is best demonstrated in a model of pulmonary infection with *F. tularensis* (24, 51) MAIT cell numbers progressively increase in the lung reaching their peak of expansion in the late phase of bacterial clearance. High MAIT cell numbers persist even after bacterial clearance, suggesting that they participate in the long-term control of infection. Interestingly, in MR1-deficient mice, not only bacterial clearance is delayed, but there is also a delay in the recruitment of conventional CD4 and CD8 T lymphocytes into the lung, indicating that MAIT cells also contribute to the establishment of adaptive immune responses. Indeed, *F. tularensis*-infected macrophages activate MAIT cells which produce GM-CSF, driving the differentiation of inflammatory monocytes into monocyte-derived DCs in the lung (51). These results show that MAIT cells are able to influence early activation and recruitment of T cells through DC maturation.

Altogether, these experimental models indicate that MAIT cells accumulate at the site of bacterial infection and are protective in various experimental infection models. However, studies in mice are not always contributive to understand the role of MAIT cells in humans, because of fundamental differences regarding their frequency and repertoire diversity. Classical mouse laboratory strains have very few, oligoclonal, MAIT cells while transgenic mice which have a high amount of monoclonal MAIT cells (52, 53). In contrast, humans exhibit high numbers of oligoclonal MAIT cells.

To date, no selective MAIT-cell deficiency has been reported in the human. Therefore, the contribution of MAIT cells to antimicrobial defense indirectly relies on correlation studies showing modifications of MAIT cell numbers in infected patients compared with healthy controls. MAIT cell frequencies are decreased in the blood of patients with various bacterial infections, including active tuberculosis (TB) (7, 11, 54–57), *Vibrio cholera* (58), and *Helicobacter pylori* (59). MAIT cell frequencies are decreased in cystic fibrosis patients with lung bacterial infections, in particular with *Pseudomonas aeruginosa*, and notably, frequencies are even lower in patients with higher inflammation and correlate with the severity of the lung disease (60). In critically ill patients with severe non-streptococcal bacterial infections, a prolonged MAIT cell depletion is associated with further development of intensive care unit-acquired infections, suggesting that MAIT cells might be protective in such a clinical setting (61). All these studies were conducted in adult patients.

As indicated earlier, MAIT cell activation can occur during various viral infections. Since viruses are unable to directly stimulate MAIT cells *via* MR1, it is likely that such MR1-independent activation is related to cytokines released from other virus-infected cells (34). In HIV infection, MAIT cells show signs of exhaustion and decline in numbers, both in the peripheral blood and gut mucosa (31, 33, 62, 63). This may leave patients particularly vulnerable to opportunistic infections. Moreover, depletion of gut mucosal MAIT cells may contribute to microbial translocation due to a compromised mucosal barrier. Upon antiretroviral treatment (ART), MAIT cells appear to be restored in the gut mucosa but not in the peripheral blood in adult patients. In HIV children, however, peripheral MAIT cells recover after ART, even more so if the treatment is started at a younger age (64). These observations support the hypothesis that the dynamics of MAIT cell peripheral expansion and tissue distribution may vary throughout life.

The case of *M. tuberculosis* infection is discussed here in more details, because it deserves particular interest for children. Indeed, the risk of rapid progression to active TB is higher in children than in adults, but in the absence of reliable biomarkers it remains very difficult to differentiate children at risk to develop active TB from those who will remain healthy and develop a latent TB infection. MAIT cells are decreased in the peripheral blood of adult patients with active TB compared with patients with latent infection and subjects without a history of *M. tuberculosis* exposure (11, 56). In addition to their low frequency, MAIT cells from patients with active TB exhibit high expression of programmed death-1 (PD-1), suggesting that they have been persistently stimulated *in vivo*, and blockade of the PD-1 pathway improves their IFNγ production in response to stimulation with a BCG vaccine (54). MAIT cells from patients with active TB have also impaired functional capacities in response to *M. tuberculosis* compared with those from patients with latent TB and healthy controls (55). Altogether, these data suggest that the degree of peripheral MAIT cell depletion correlates with disease outcome. However, MAIT cell frequencies show a high variability between individuals (healthy controls as well as infected patients), making it unlikely to use them as a clear-cut biomarker of disease outcome, unless longitudinal studies in large cohorts of patients provide convincing results.

It is usually proposed that the reduced MAIT cell numbers in the peripheral blood of infected patients is a consequence of their recruitment to the infected tissues. However, data on MAIT cell accumulation in the tissues remain controversial, owing to the difficulty to perform longitudinal studies in patients. Thus, MAIT cells are detected in the lungs of patients with active TB (7, 11). At contrast, MAIT cell frequencies are reduced in pleural effusions, but increased in ascitic fluids from patients with tuberculous peritonitis, suggesting that MAIT levels may vary depending on the tissues (54). So far, one cannot exclude that a low frequency of MAIT cells in some individuals may, by itself, favor bacterial colonization and promote disease progression. As recently shown, a polymorphism in the human MR1 gene, associated with MR1 expression, is associated with susceptibility to meningeal TB in Vietnamese adult patients (65). It will be crucial to know if such association is observed in other microbial infections in various populations, to determine if impaired MR1-antigen presentation is involved in susceptibility to infection.

## How MAIT Cells Distinguish Pathogens from Commensals?

Because MAIT cells are activated in the presence of microbial-derived MR1 ligands and are able to kill infected cells, their activation must be tightly controlled to avoid inappropriate responses to commensals. This is particularly crucial in mucosae (gut and lung) where MAIT cells are abundant and in close vicinity to the microbiota. Several lines of evidence indicate that MAIT cells can adapt their proliferative and effector responses depending on the amount, nature, and location of microbial ligands, and on the presence of co-stimulatory signals (66–69). Thus, MR1-mediated presentation of microbial ligands may not be sufficient to optimal MAIT cell responses. MR1 transcripts are detected in multiple tissues, but MR1 expression at the cell surface is very low in the absence of infection. MR1 is retained in the endoplasmic reticulum until ligand binding occurs, at which time it is rapidly transported to the cell surface (70). In antigen-presenting cells, uptake of intact bacteria is required for efficient MR1-mediated MAIT cell activation, while stimulation with soluble ligand is inefficient. In addition, the amount of MR1 at the cell surface is differentially regulated in different cell types. Toll-like receptor (TLR) stimulation may modify MR1 expression on antigen-presenting cells and B cells (71, 72). In mice, the administration of the synthetic MR1 ligand 5-OP-RU alone causes MAIT cell activation but does not result in MAIT proliferation, while the addition of TLR agonists causes high levels of activation and proliferation of the MAIT cell pool (50). Altogether, these data may explain why the mere presence of commensal-derived ligands is not sufficient to induce surface MR1 expression and MAIT cell activation in the gut. It is likely that a high infiltration of pathogen bacteria, due to the disruption of the intestinal barrier, together with strong inflammatory signals, is required. Moreover, compared with peripheral blood, MAIT cells from mucosae have increased expression of certain genes, such as *TNF, IL23R*, and *CD40L*, that would allow them to respond quickly to bacterial infiltration (69). Finally, active compounds produced by some commensals may maintain a state of suppression of gut MAIT cells, as suggested by the decreased IFNγ production by MAIT cells in response to *Staphylococcus aureus* stimulation if commensal *Lactobacilli* bacteria are present (73). The production of folic acid by *Lactobacillus plantarum* was involved in the maintenance of regulatory T cells (74). Interestingly, the folic acid derivative, 6-FP, is able to bind MR1 but acts as an antagonist ligand for MAIT cell activation (17). Whether *L. plantarum* has an intact riboflavin biosynthetic pathway able to produce activating MR1 ligands that compete with 6-FP remains an open issue. The recent success of a synbiotic trial associating *L. plantarum* to fructooligosaccharides to prevent sepsis in rural Indian newborns paves the way for such investigation (75).

## Conclusion

Our knowledge of MAIT cells and their role in the control of microbial infections has grown substantially in the recent years. In humans, numerous studies were conducted in adults, but studies regarding MAIT cell function in children are still lacking. Whether blood MAIT cell frequency could be used as biomarker for disease outcome requires further investigation in longitudinal cohorts. A better knowledge of MAIT cell interactions with pathogens and cross talk with other immune cells will also be crucial for the development of new therapeutic or vaccine strategies to prevent the development of infectious diseases.

## Author Contributions

All authors listed have made a substantial, direct, and intellectual contribution to the work and approved it for publication.

## Conflict of Interest Statement

The authors declare that the research was conducted in the absence of any commercial or financial relationships that could be construed as a potential conflict of interest.
